# Mindfulness in medical education: coming of age

**DOI:** 10.1007/s40037-020-00598-w

**Published:** 2020-07-06

**Authors:** Ronald M. Epstein

**Affiliations:** Family Medicine, University of Rochester Medical Center, Rochester, NY USA

The word ‘mindfulness,’ relatively unheard-of in medical education before 1998, has become a guiding principle of some thoughtful efforts to promote reflection, self-awareness and well-being in pre- and post-graduate and continuing medical education. Mindfulness refers equally to attentive focus and adapting to the unexpected in the operating room as it does to conducing a family meeting regarding a dying patient. While the emphasis on mindfulness is welcome for those entering and practicing an increasingly beleaguered profession, the term has also become popularized, conflated with particular techniques and practices, and associated with claims that range from the sensible to the sensational. There is also confusion about what ‘being mindful’ actually is, reflected in the divergent and heterogeneous educational programs claiming to cultivate it [1, 2].

Mindfulness, in my view, is not a ‘thing’ but rather a vector; it is dynamic and heading toward goals that are pragmatic and ethically robust. In fact, the origins of the word ‘mindfulness’ are within the context of a philosophical frame that emphasizes ‘right mindfulness’ as one of eight principles for living a righteous and transformative life. Mindfulness is not the same as reflection. While reflection often refers to activities undertaken after-the-fact (what the educator Donald Schön called ‘reflection on action [3]’), mindfulness is more akin to Schön’s ‘reflection in action’—it is a practice in the moment. Mindfulness in medicine includes the capacity for non-judgmental attention to self and other *in the moment *during every day work—and with the goal to act with clarity, resolve, compassion, practical wisdom and interpersonal effectiveness [4]. Particularly important are the qualities of presence, curiosity and beginner’s mind—the capacity to see a familiar situation with new eyes and hold two or more contrasting perspectives at the same time without the need for premature resolution [5]. Leaving those skills for discussion in rounds the next day is helpful, but not enough. In these times of pandemic virus, gut-wrenching angst and epistemic uncertainty, the need for moment-to-moment clarity, tolerance of ambiguity, emotional intelligence and ethical awareness—framed by an ability to hold contradictions and inconstancies in information and guidance—is greater than ever. Moreover, our responsibility as medical educators is to make every effort to help our students and junior colleagues acquire those capabilities.

The clarity of intent and methods in the Show and Tell in this issue by Tom Hutchinson and Stephen Liben about their novel curricular elements at McGill University are particularly welcome [6]. Their program addresses important issues such as the relationship between self-awareness, well-being and clinical effectiveness (including empathy, communication and clinical decision-making); they build on successes and failures of prior attempts to humanize medical training; they have thought carefully about timing and format so that it would have maximal impact; and they introduce a philosophical intersection between an influential thinker in psychology (Virginia Satir) and principles of mindfulness. I look forward to their evolving reports of effectiveness as well as challenges, as these will guide other institutions’ efforts.

Drs. Hutchinson and Liben raise some important challenges to doing this work, challenges that our mindful practice group at the University of Rochester has also faced. First, can you actually *teach* someone to be mindful, reflective and self-aware? Or, rather, do you till the soil, provide water and nutrients, pull a few weeds, and optimize the conditions under which those qualities can grow? Rollo May, another mid-20th-century psychologist, once said that ‘*human freedom involves our capacity to pause between stimulus and response and, in that pause, to choose the one response toward which we wish to throw our weight *[7]*.*’ In medicine, the stimuli happen quickly and often under duress—a cardiopulmonary resuscitation, an angry patient, a grieving clinician, an unanticipated turn of events. The qualities of mindfulness, humanism, empathy, clarity and pragmatism must suffuse *that *moment in order to inform responses that are concordant with what we conceptualize as best practice. Like many aspects of medical expertise, this is a lifetime of work, not merely a course.

Second, how do you know when someone is practicing mindfully? Validated scales of mindfulness [8] might be useful; they appear to correlate with other things that matter, such as empathy and compassion, patient-centeredness, patient safety, implicit bias and communication [9, 10]. No matter how valid the scales might be, though, only a trained observer can determine whether those skills and qualities are applied appropriately and ethically—does this student not only know the words of empathy, but also apply them at the right moment, and equitably? There is no substitute for careful observation, both in real or simulated environments, and by their very nature, assessments based on careful observation are always intersubjective, contextual and evolving [11]. Outcomes also have to be clearly defined, and realistic.

Third, who can and should teach skills of mindfulness, self-awareness and reflection? Here, commitment to self-awareness in one’s own life is fundamental, whether through daily contemplative practice or other means. Self-awareness, and mindfulness, are more than personal qualities, they are habits, and those habits become part of who we are. Transparency about how we, as teachers, have learned and sustain those habits can be enormously instructive to learners at every level. Imparting information and being the expert is important at times, but equally important are the skills of deep listening, small group process, practical wisdom and embodiment. Such teachers need training and ongoing mentoring and the medical education enterprise needs to invest in ongoing faculty development.

Mindfulness-oriented medical educational programs are becoming mainstream and now face the test of applying them in the messy realities of clinical training. We should be careful to be aware of the promises as well as the challenges in moving forward, and not expecting too much, or too little, from efforts such as those at McGill and elsewhere. One outcome of these efforts—at McGill and also at Rochester, and not part of the stated intent—has been a greater sense of community [12]. Recent changes in medicine have made physicians feel more isolated from one another [13], further exacerbated by the physical distancing and work-from-home required by the current pandemic. Fostering a greater sense of collective mindfulness, community and solidarity among physicians facing formidable times is both powerful and empowering.
